# Assessment of Emergency Responders After a Vinyl Chloride Release from a Train Derailment — New Jersey, 2012

**Published:** 2015-01-09

**Authors:** Kimberly Brinker, Margaret Lumia, Karl V. Markiewicz, Mary Anne Duncan, Chad Dowell, Araceli Rey, Jason Wilken, Alice Shumate, Jamille Taylor, Renée Funk

**Affiliations:** 1 Epidemic Intelligence Service, CDC; 2 National Institute for Occupational Health and Safety, CDC; 3 New Jersey Department of Health; 4 Agency for Toxic Substances and Disease Registry

On November 30, 2012, at approximately 7:00 am, a freight train derailed near a small town in New Jersey. Four tank cars, including a breached tank car carrying vinyl chloride, landed in a tidal creek. Vinyl chloride, a colorless gas with a mild, sweet odor, is used in plastics manufacture. Acute exposure can cause respiratory irritation and headache, drowsiness, and dizziness; chronic occupational exposure can result in liver damage, accumulation of fat in the liver, and tumors (including angiosarcoma of the liver) (1). Because health effects associated with acute exposures have not been well studied, the New Jersey Department of Health requested assistance from the Agency for Toxic Substances and Disease Registry (ATSDR) and CDC. On December 11, teams from these agencies deployed to assist the New Jersey Department of Health in conducting an assessment of exposures in the community as well as the occupational health and safety of emergency personnel who responded to the incident. This report describes the results of the investigation of emergency personnel. A survey of 93 emergency responders found that 26% of respondents experienced headache and upper respiratory symptoms during the response. A minority (22%) reported using respiratory protection during the incident. Twenty-one (23%) of 92 respondents sought medical evaluation. Based on these findings, CDC recommended that response agencies 1) implement the Emergency Responder Health Monitoring and Surveillance (ERHMS) system (2) for ongoing health monitoring of the emergency responders involved in the train derailment response and 2) ensure that in future incidents, respiratory protection is used when exposure levels are unknown or above the established occupational exposure limits.

The CDC team created a self-administered survey based on the ATSDR toolkit for the assessment of chemical exposures (3) to assess health effects, use of personal protective equipment, and preparedness training among emergency responders who worked at the incident site at any time during November 30–December 7, 2012. The CDC team met with emergency response leaders and local responders during the period December 11–21. Emergency responders completed surveys during the meetings, and those who did not attend any meetings had the option of mailing in a survey; 93 completed surveys were received.

Responders were categorized by profession, including emergency medical services, firefighters, police officers, and hazardous material technicians, and by cumulative duration of exposure. Because a typical work shift lasts 12 hours, participants were categorized as working ≤12 hours or >12 hours at the incident site during the entire 8-day period.

Symptoms were grouped according to clinical presentation (i.e., neurologic [dizziness, weakness, and loss of balance], upper respiratory [runny nose, burning nose or throat, and hoarseness], and lower respiratory [shortness of breath, chest tightness, wheezing, and burning chest sensations]). Coughing, increased congestion, and increased phlegm are presented separately from other respiratory indicators because their causes could be upper or lower respiratory in nature. Headache; nausea and vomiting; irritation, pain, or burning of the eyes and skin; and diarrhea were also reported.

Use of personal protective equipment, including respiratory protection, was assessed as well. In addition, respondents were asked questions to evaluate preparedness training. A bivariate analysis was conducted using statistical software.

A total of 93 surveys were completed, though not all questions were answered in all surveys. Of these, 72 were completed during meetings with emergency response leaders and local responders, and 21 were mailed in at a later time. Ninety-six percent of respondents were male and white, and the median age of respondents was 42 years (range = 19–78 years). Forty-eight percent (44 of 92) of respondents reported spending >12 hours at the site, and 52% (48 of 92) reported spending ≤12 hours at the site.

The most frequently reported symptoms were headache (26%), upper respiratory symptoms (26%), and lower respiratory symptoms (22%) (Table 1). Other symptoms reported included coughing; neurologic symptoms; nausea and vomiting; congestion or phlegm; irritation, pain, or burning of the eyes; irritation, pain, or burning of the skin; and diarrhea (Table 1). The prevalence odds ratios for lower and upper respiratory symptoms; irritation, pain, or burning of the eyes; and headache were significantly associated with an exposure >12 hours (Table 2).

Twenty-three percent (21 of 92) of respondents reported wearing no personal protective equipment (Figure). When asked a separate question about respirator types, 20 respondents (22%) reported donning a self-contained breathing apparatus during the response, although it is unclear when respiratory protection was used during the response. Of these 20 respondents, one was an emergency medical services worker, one was a police officer, two were hazardous material technicians, and 16 were firefighters. One reported using both a self-contained breathing apparatus and a powered air-purifying respirator, another reported using a full-face air-purifying respirator, and one reported using an air-purifying respirator but did not specify which type. Forty-nine percent (35 of 72) of respondents who reported they did not wear respiratory protection on initial arrival at the site stated that respiratory protection was not required for their work, 24% (17 of 72) stated none was available, 17% (12 of 72) stated they were not advised to wear respiratory protection, and 17% (12 of 72) stated they did not think they needed it. Eight percent (six of 72) of respondents reported they were told respiratory protection was not necessary, and 1% (one of 72) stated that it got in the way of work. Categories are not mutually exclusive.

## Discussion

The Occupational Safety and Health Administration (OSHA) permissible exposure limit for vinyl chloride is 1 part per million, based on an 8-hour time-weighted average (4). CDC recommends reducing vinyl chloride exposures to the lowest feasible concentration because it has been designated a potential occupational carcinogen (5). According to OSHA regulations, employees engaged in emergency response who have potential exposures to hazardous substances should wear a positive pressure respirator until the incident commander determines (through the use of air monitoring) that a decreased level of respiratory protection will not result in hazardous exposures to employees (6). During the emergency response described in this report, exposure monitoring was unavailable, and the majority of respondents did not use respiratory protection. The need for respirators and selection of particular respirator types are determined by an exposure risk assessment. The implementation of a respiratory protection program, including the use of exposure monitoring to determine when respirator use is required, might assist emergency responders in future events.

Symptoms were commonly reported by first responders, most frequently headache, upper respiratory irritation, and lower respiratory irritation. Because personal breathing zone measurements of responders’ exposures to vinyl chloride were not collected, it is impossible to correlate vinyl chloride exposure levels with symptoms. On the basis of the OSHA and CDC guidance described previously, respiratory protection would likely have been required for many first responders. Proximity to the evacuation zone and assigned job task could be used as proxy indicators of the need for respirator use.

What is already known on this topic?

Vinyl chloride, a gas used to make plastics, is an acute respiratory irritant that can cause headache, drowsiness, and dizziness. Chronic exposure can damage the liver.

What is added by this report?

In December 2012, vinyl chloride was released from a breached tank car after a train derailment in New Jersey. A survey of 93 emergency responders found that 26% experienced headache and upper respiratory symptoms during the response. Only 22% reported using respiratory protection during the incident, and 23% sought medical evaluation. Most respondents reported having received some emergency responder training and felt they had sufficient instruction, indicating a possible gap in perception of risk.

What are the implications for public health practice?

In similar incidents, health officials are encouraged to implement a framework for health monitoring and surveillance of emergency responders, encourage use of respiratory protection until engineering controls and work practices can be implemented that reduce exposure to below the appropriate occupational exposure limit, and evaluate training needs for all emergency response roles.

The findings in this report are subject to at least three limitations. First, complete rosters of emergency responders who worked in the evacuation zone and the period over which work shifts occurred were unavailable; therefore, the study was lacking a strong denominator. Selection bias likely occurred because the sample consisted of emergency responders who attended the scheduled meetings and completed the survey there or who obtained surveys from emergency response leaders and mailed them in; an accurate account of responders who arrived on the scene is not available so it is possible that there were a number of emergency responders who could not attend the meetings or were never given a survey. Second, personal breathing zone measurements of responders’ exposures to vinyl chloride were not collected, so it is impossible to correlate vinyl chloride exposure levels with symptoms. Finally, the small number of participants who completed the survey made it impossible to meaningfully analyze the associations between respirator use and symptoms.

For ongoing health monitoring of the emergency responders involved in the train derailment response, and to prepare for future incidents, the response agencies involved should consider implementing the ERHMS system, a framework that includes recommendations and specific tools to protect emergency responders during all phases of a response, including pre-deployment, deployment, and post-deployment. Respiratory protection should be used until engineering controls and work practices that reduce employees’ exposures to below the appropriate occupational exposure limit (OSHA-permissible exposure limit or CDC-recommended exposure limit) can be implemented. Implementation should follow the OSHA respiratory protection standard (7). A positive pressure respirator should be used when exposure levels are unknown and until they have been determined to be below the appropriate occupation exposure limit. Furthermore, the authority having jurisdiction and the various emergency response departments can refer to existing National Fire Protection Association standards (8).

## Figures and Tables

**FIGURE:**
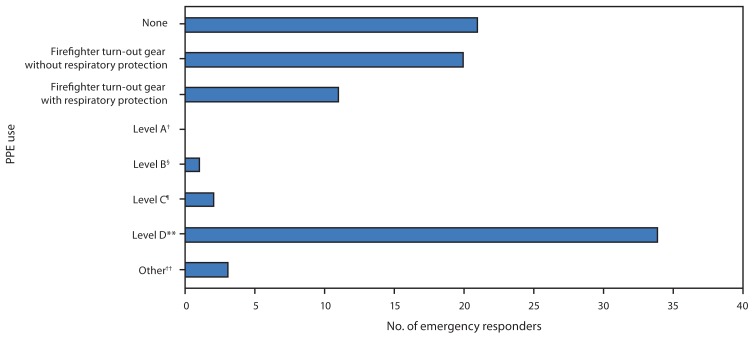
Personal protective equipment (PPE)* use among emergency responders (N = 92) after a vinyl chloride release from a train derailment — New Jersey, 2012 * Information on PPE levels and risks available at https://www.cseppportal.net/csepp_portal_resources/ppe_factsheet.pdf and http://www.cdc.gov/niosh/docs/2008-132/pdfs/2008-132.pdf. ^†^ Level A: Recommended when greatest potential for exposure to skin and respiratory system exists. Includes a pressure-demand, full face-piece; a self-contained breathing apparatus; and a fully-encapsulating, chemical-resistant suit. ^§^ Level B: Recommended when highest level of respiratory protection is indicated but skin at a lesser level. Includes a pressure-demand, full face-piece; a self-contained breathing apparatus; and chemical-resistant clothing. ^¶^ Level C: Recommended when concentration or type of substance is known and criteria for respiratory use are met. Includes a full face-piece or half-mask; an air-purifying, canister-equipped respirator; and chemical-resistant clothing. ** Level D: Recommended when minimum protection is required. Includes a simple work uniform. ^††^ Other forms of PPE include coveralls, gloves, safety glasses, composite-toed shoes, and hard hats.

**TABLE 1:** Self-reported symptoms of emergency responders (N = 93) after a vinyl chloride release from a train derailment — New Jersey, 2012

Symptom*	No.	(%)
Headache	24	(26)
Upper respiratory	24	(26)
Lower respiratory	20	(22)
Coughing	15	(16)
Neurologic	14	(15)
Nausea or vomiting	14	(15)
Increased congestion or phlegm	11	(12)
Irritation, pain, or burning of eyes	11	(12)
Other	3	(3)
Irritation, pain, and burning of skin	2	(2)
Diarrhea	1	(1)

* Symptoms are not mutually exclusive.

**TABLE 2:** Odds of reporting selected symptoms, by hours worked in evacuation zone (>12 hours versus ≤12 hours), among emergency responders (N = 93) after a vinyl chloride release from a train derailment — New Jersey, 2012

Symptom*	Prevalence OR	95% CI
Lower respiratory	14.1	3.0–135.0
Irritation, pain, or burning of eyes	5.8	1.1–58.6
Upper respiratory	3.9	1.3–13.9
Headache	3.6	1.2–11.8
Coughing	3.2	0.8–15.2
Neurologic	3.2	0.8–15.2
Increased congestion or phlegm	2.8	0.6–18.0
Nausea or vomiting	2.2	0.6–9.1

**Abbreviations:** OR = odds ratio; CI = confidence interval.

* Symptoms are not mutually exclusive.
